# Are Farmers in National Park Communities Willing to Reallocate Their Lands? A Situational Analysis

**DOI:** 10.3390/ijerph19148589

**Published:** 2022-07-14

**Authors:** Yan Gao, Qian Dong, Yi Deng

**Affiliations:** School of Tourism and Hospitality Management, Hubei University of Economics, Wuhan 430205, China; ygao@hbue.edu.cn (Y.G.); ydeng@hbue.edu.cn (Y.D.)

**Keywords:** community farmers, land reallocation intentions, Shennongjia National Park, structural equation model, situational analysis

## Abstract

Limited by China’s mixed land ownership model, which is divided into collective and state ownership, national parks’ strict ecological protection measures of restricting land use patterns and intensity are subject to the decisions made by collective landowners and contract operators, namely, rural households in national park communities. The disposition and intention of community farmers regarding collective land ownership is related to the nature conservation effect of the national park. In the context of national park land functions for ecological conservation, environmental education, leisure and recreation, scientific research, and “nest eggs” (basic living guarantees), the research on the influencing factors of farmers’ intentions to reallocate their land (expropriated or transferred) will provide a basis for a National Parks Administration (NPA) to develop supporting policies for collective land reallocation in different functional zones and to prevent community conflicts. The research took Shennongjia National Park as an example and, combined with literature analysis, used the Structural Equation Model (SEM) to explore the influencing factors of community farmers’ land reallocation intentions and drew the following conclusions: farmers’ intentions to leave their land for nature conservation purposes and for urbanization purposes are different. In the five land function situations above, farmers’ perceptions of land function in national parks did not directly affect their land reallocation intentions, while their trust in the land management ability of NPA was a complete mediator. Farmers’ preferences for the economic value of land had no significant moderating effect on land reallocation intentions. Farmers’ characteristics have a moderating effect on different land function situation models. Older and less educated farmers are more likely to receive livelihood compensation rather than monetary compensation after leaving their land. Therefore, some management suggestions are put forward, such as strengthening the capacity for building national park land and other natural resources management, adapting to the collective land policy in different function zones, and paying attention to the livelihood compensation of community farmers after they leave the land.

## 1. Introduction

Establishing a new natural reserve system with national parks as the main body was proposed by the report of the 19th National Congress of the Communist Party of China (CPC) in 2017, noting that China’s natural reserve system construction has entered the era of national parks. Land protection measures must be sensitive to the rudiments of these land tenure arrangements. National parks fulfill the important function of protecting national ecological security through strict restriction of land use mode and intensity. The ownership of natural resources such as land directly affect the protection effect [1,2,3,4]. Collective Land Ownership and State Land Ownership are the two most dominant forms of land tenure in China and are similar to the complex mosaic of land tenure of rangelands in the United States and other countries [5]. Collective Land Ownership means that the ownership of the land belongs to the village collective, but the farmer households have the contract and management rights to the land and are the actual controllers of the collective land.

If strict protection measures are implemented on the premise that all land under protection is owned by the state, the resistance to strict protection will undoubtedly be greatly reduced. However, this is not the reality. Strategies to amalgamate China’s Protected Lands into the national parks structure face dichotomous difficulties. As most lands are under Collective Ownership and the large numbers in the indigenous population [6], strict land management protection measures are bound to be counterproductive and encounter resistance under these conditions of diverse land tenure.

The Overall Plan for the Establishment of a National Park System (2018) calls for “*ensuring that natural resource assets owned by the state occupy a dominant position and are managed with feasibility*”. This is to be realized using three distinct strategies, covering the following:

1. Creating a unified, standardized, and highly efficient National Park Management Structure/System,
2. Prioritizing protection of ecological and natural assets, and
3. Prioritizing public and social welfare.

This tripartite objective will result in two important transformations in land management within the new national parks structure. Firstly, the state will expropriate all Collective Lands, and secondly, according to Wang, et al. (2014) [7], it will undertake stringent land use policies that may potentially deprive local communities of important extended living spaces. Under the circumstance that the natural ecosystem and the community in the protected area have been deeply interbedded in China, if all the land is “universally” acquired by the state, although conducive to the realization of the goal of the “unified, standardized, and highly efficient” treatment of the national park, it is not feasible in management. The reasons are as follows.

First, the “one size fits all” expropriation of these lands limits the space for community development, essentially depriving community residents of their sources of livelihood [8]. When an alternative livelihood is not replaced in time, it is easy to cause community conflicts, possibly leading to the destruction of the ecological environment in the community [9]. This has been learned from previous “isolated island” protection practices. Therefore, whether this action can promote the improvement of the efficiency of ecological protection is still debatable. Second, gaps created between the implementation of the national park structure and the possible disruptions in the traditional community socio-economic and cultural activities that are aligned to the land will need to be filled early in the process. It is necessary to defend and protect traditional cultural and social practices. Finally, there are concerns regarding the move of establishing the National Parks System and its ability to eventually promote ecological conservation. This is especially disquieting since there will the need for legislation to expropriate collective lands by the state, which is likely to require some sort of compensation involving land tenure arrangements. This will undoubtedly cause significant pressure on the state’s economic resources, due to the sheer number of people involved [10]. Land compensation also places great economic pressure upon the government [8], so the exploration of community protected areas is also required [11]. Accordingly, how to deal with collective land ownership has become an important issue in the pilot process of the national park system.

The Guidance on Establishing a Nature Reserve System with National Parks as the Main Body (2019) was published jointly by the General Office of the CPC Central Committee and the State Office. The Guidelines proposed by the report postulate that, given the principles of volunteerism and compensation, the state should explore strategies to safeguard the rights and interests of property owners. This should also involve approaches that realize the diverse protectionist policies of the collective land tenure arrangements and the social and cultural dynamics of the indigenous and rural populations. These protectionist policies are proposed to be realized in the various natural protected areas by means of lease, relocation and replacement, purchase and cooperation. Formulating strategies unique to each functional zone will eliminate the “one size fits all” approach and leave room for better land management. According to the current functional zoning of national parks, strictly protected areas adhere to the state ownership of land and the orderly expropriation of collective land; the collective land in the general control area is allowed to explore various land circulation methods, such as leasing and redeeming, while the National Park Administration (NPA), on behalf of the state, is responsible for obtaining the management (protection) easement for all the land, limiting the extensive land use mode and intensity [8].This diversified land ownership model not only strictly implements the principle of the national park ecological protection first but also takes into account the needs of community development, so it is a feasible way to solve community conflicts.

In this research, collective land expropriation, transfer, lease, replacement, and other forms of abandoning the original land use and intensity are defined as “land reallocation”. In all forms of land reallocation, the national park will enforce the conservation easement of all collective land to ensure that the use of collective land will not be abused after reallocation to a third party [12]. Of the 10 national parks areas currently across China, approximately 50% have at least one third of the land falling under some Collective Land Ownership regime. As such, the interest of farmers whose land tenure is classified as unclear and collective must be strategically considered, whether their lands are expropriated by the state or allowed to be transferred, to minimizes the farmers’ displacement and disenfranchisement. The NPA must also consider the attitude and perception that the farmers, who are under Collective Land Ownership, have towards land expropriation and relocation. The management of these farmers will determine the overall success of the national parks project [7]. Detecting the willingness and overall attitude of farmers under Collective Land Ownership to abandon their rights to land will undoubtedly complicate the process of establishing China’s national parks project. Consequently, there is a need to determine the factors influencing land surrender and reallocation intentions. Determining the responses to these and other Collective Land Ownership concerns will be an important basis for the formulation of policy and planning for the establishment of national parks. Resolving these concerns will reduce potential community conflicts and foster farmers’ willingness to participate in the project. At the same time, the current academic research on willingness to leave land and its influencing factors almost all focus on the research on land reallocation intentions from the perspective of rural labor transfer or the economy under the background of urbanization [13,14]. In the context of protected nature areas, research on farmers’ willingness to leave land is relatively lacking in situations where the functions of national park land are for ecological protection, environmental education, scientific research, leisure and recreation, “nest eggs”, and so on.

In conclusion, it is an urgent task to clarify the land reallocation intentions of rural households in national park communities under different land function situations. Therefore, the research took Shennongjia National Park as an example, using questionnaire and semi-structured interviews to explore community farmers’ land reallocation intentions and their influencing factors in national parks under different land function situations. We use the Structural Equation Model (SEM) method to investigate the mechanism and causal relationship between farmers perceptions of park land function, farmers’ trust in park land management abilities, farmers’ land economic value preferences, household characteristics, and land reallocation intentions. The research results provide a scientific, reasonable, and effective basis for the Collective Land Ownership disposition of the national parks.

## 2. Literature Review and Research Hypothesis

### 2.1. Research Hypothesis

The research attempts to answer three questions: first, what are the factors that influence farmers’ land reallocation intention (LRI) under different situations of land functions in national parks? The integrated approaches of a literature review and the understanding of the reality of China were used to find out the influencing factors of farmers’ land reallocation intentions in national parks. Community farmers’ perceptions about the land function of national parks, land management ability of NPA, characteristics of farmers, and farmers’ preferences of land economic value were taken as influencing factors for land reallocation intention. Second, how do the influencing factors affect the land reallocation intention? The Structural Equation Model (SEM) is used to reveal the mechanism of action. Third, is there a difference in the land reallocation intentions for the purpose of urbanization vs. that for nature conservation? A comparative study was applied to infer the differences of farmers’ land reallocation intentions against the background of nature conservation and urbanization.

Therefore, based on practical experience and existing research literature, this study proposed the following hypotheses:

**Hypothesis** **1** **(H1).** *Community farmers’ perceptions of national park land function (PNPLF) affect their land reallocation intention (LRI).*

According to cognitive behavioral theory, farmers’ land reallocation intentions are affected by their perception of land functions [15,16]. Land function is different from land value [17], but a large amount of the literature does not clearly distinguish between land function and land value. This research considers that land value is the economic manifestation of land function. To some extent, land value assessment can urge landowners to pay attention to ecological protection, scientific research, cultural carriers, and other functions of land. Farmers’ land values have a certain degree of influence on land reallocation intentions. Wang et al. (2018) hold that the consistent relationship between farmers’ cognition and behavior regarding farmland ownership adjustment is an important content of theoretical research on farmland ownership adjustment [18]. There is also much research on the relationship between land function perception and land reallocation intention. For example, Xu (2014) studied the relationship between land function preferences and farmland reallocation and proved that farmers’ different preferences for land functions had different degrees of influence on the transfer intention [19].

Land functions are expanding with the development of society and the change of demand. Agricultural land had multiple functions [20]. At the practical level, the function of farmland depends on the nation demand for land use. Land function is situational, and different land use situations determine the various land functions. Therefore, the land function of national parks in China is different from general agricultural land. The primary function of farmland is mainly a supply function, including a production function and carrier function. The former refers to food production and cash crop planting, while the latter mainly refers to the carrying of traditional culture and values, which is a non-economic factor [21]. In the context of China, as the land has been dominated by farming culture since ancient times, land is also the source of livelihood for farmers and the basic guarantee for their pension, employment, medical care, and life necessities [22]. Therefore, agricultural land has the “nest egg” function, which means a basic living guarantee. The land ownership policy of national parks cannot deprive farmers of basic living security, and the land “nest egg” function still needs to be realized within the scope of national parks. Moreover, according to the Guidelines for the National Park Function Zoning, which is a Forestry Industry Standard of the People’s Republic of China (LY/T2933-2018), the functional zoning of national parks is divided into strictly protected zones, ecological conservation zones, traditional utilization zones, and environmental education zones. Consequently, land functions of national parks should also include ecological conservation, recreation, scientific research, environmental education, and so on. To sum up, the hypothesis is proposed as follows: land functions that include the ecological conservation function (ECF), nest egg function (NEF), leisure and recreation function (LRF), scientific research function (SRF) and environmental education function (EEF) will affect the land reallocation intention (LRI).

**Hypothesis** **2** **(H2).** *Farmers’ trust in the land management ability (TLMA) of the national parks administration is the mediating factor between the perception of land function and land reallocation intention*.

Studies have shown that farmers’ willingness to transfer land will be affected by the credibility of the government. Farmers with high trust in the government have higher willingness to transfer land. For example, Wang et al. (2017) and Pu et al. (2018) concluded through case studies that farmers with high trust in the government have higher willingness to transfer agricultural land [23,24]. On the premise of high trust in the government, the probability of mass conflicts in the process of land expropriation will be low [25,26]. The failure of government behavior to meet public psychological expectations is the main reason for reducing government credibility [27]. The promotion of management or governance ability to credibility has been verified in relevant studies [28].

In the context of national parks, the NPA manages and operates the national parks on behalf of the central government, and its management ability is one of the main factors affecting credibility. When the land management ability of NPA is not sufficient to meet the expectations of community farmers, or the community farmers are full of doubts about the land management ability of NPA, this is likely not only to affect the community farmers’ trust in NPA but also the farmers’ land reallocation willingness. Therefore, TLMA is taken as a mediator, and we discuss its influence mechanism on LRI.

**Hypothesis** **3** **(H3).** *Farmers’ land preferencse for economic value (PEV) in the national park have a moderating effect on LRI.*

The preference for the economic value of land will lead to large differences in farmers’ willingness to relocate from land [16]. Based on the survey data of farmers in Shuyang County, Jiangsu province, Zhao et al. (2012) concluded that the direct economic value of land (grain production, etc.) was negatively correlated with farmers’ willingness to leave their land, while the indirect economic value, such as the expectation of land transfer (rent per unit area), was positively correlated with their land reallocation intention [15]. Yang et al. (2013) conducted a questionnaire survey among rural households in suburban villages and suburban villages in Hongta District, Yuxi City, Yunnan, China and found that rural households’ awareness of land compensation function was lower than that in suburban villages, which would affect farmers’ willingness to transfer land to some extent [29]. Xu (2014) conducted an empirical study on peasant households’ willingness to transfer land in developed and undeveloped regions and proved that peasant households in developed regions preferred the property function of land, and the economic value of land was relatively high [19]. The land reallocation could bring them higher economic income and guarantee their living standards. Therefore, peasant households in developed regions had a strong desire to leave their land. However, farmers in undeveloped areas prefer the land production function, and the economic income brought by land reallocation is not high, so the land reallocation intention in undeveloped areas is low [16]. Previous research has shown that the economic development level will affect farmers’ cognition of the land’s economic function. When land brings considerable indirect economic income, such as rent and compensation, farmers tend to transfer land and have a stronger desire to reallocate land. However, in the case of natural ecological protected areas, how farmers’ preference for the economic value of land affects their willingness to leave the land remains to be verified. Therefore, this study proposes the hypothesis that farmers’ land economic value preference has a moderating effect on land reallocation intention in national parks.

**Hypothesis** **4** **(H4).** *The household characteristics (HC) of the national park community have a moderating effect on the LRI.*

The research defines the characteristics of farmers as individual characteristics and family characteristics [30]. In the existing literature on farmers’ reallocation intention, individual factors of farmers include their education level, age, and gender [30,31]. The type of landowner is related to the way the land is used [32], and thus the characteristics of the farmers must be considered. The characteristics of peasant households include household resources (whether they have off-farm employment skills) [15,31] and the percentage of agricultural income making up their total income [33]. The higher the proportion of agricultural income in the total household income, the lower the willingness to engage in land transfer [16]. Farmers’ willingness to leave land is closely related to off-agricultural employment to a large extent [15].

In conclusion, combined with the national park context, the research proposes the hypothesis that the characteristics of farmers in the national park community have a moderating effect on farmers’ willingness to leave the land. Characteristics of farmers include age, education, household income, and off-farm employment skills.

### 2.2. Theoretical Model Construction

**Figure 1:**
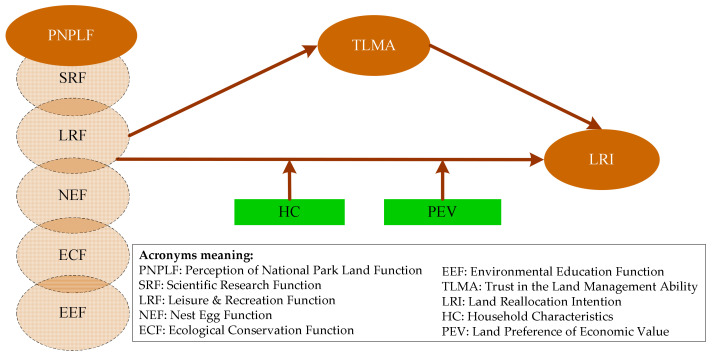
The conceptual model.

Based on the four research hypotheses proposed above, we constructed a theoretical model of influencing factors of farmers’ land reallocation intention in national parks (Figure 1). Although the theoretical model is based on the research results of influencing factors of farmland ownership adjustment under the background of rapid urbanization, it also considers the land function demands of national park ecological protection, leisure and recreation, scientific research, environmental education, basic living guarantees, and the role of farmers’ trust in the land management ability of NPA.

## 3. Overview of the Research Area

**Figure 2:**
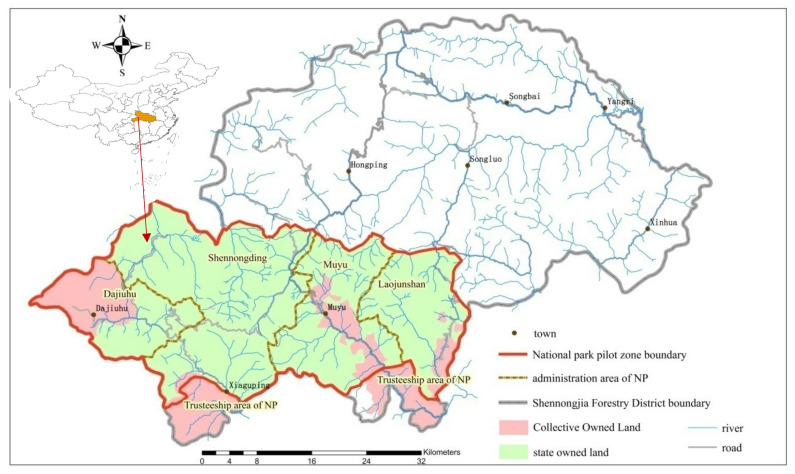
Land ownership of Shennongjia National Park.

The system pilot area of Shennongjia National Park, which is also a World Natural Heritage Area, is located in the southwest of Shennongjia Forest district, Hubei province, covering an area of 1169.88 km^2^, accounting for 35.97% of the total area of Shennongjia Forest district. The national park includes Jiuhu Town, Xiaguping Town, Muyu Town, Hongping Town, and Song Luo township. According to the General Plan of Shennongjia National Park (hereafter referred to as The Plan), the state-owned land area of the park system pilot area is 1005.79 km^2^, and the collective land area is 164.09 km^2^. During the system pilot period (2016–2020), the southern land of Shennongjia Forest district was entrusted to Shennongjia National Park. After the end of the pilot period (2021–2025), the trust area will be officially included in the Shennongjia National Park. The total area of the national park will be increased to 1325.06 km^2^, of which the collective land area will be increased to 307.37 km^2^, accounting for 23.2% of the total area of the national park. The general situation of land ownership in Shennongjia National Park is shown in Figure 2. During the vision planning period (2026–2030), the area of the park will be extended to the whole Shennongjia area, and Hubei Padong Golden Monkey National Nature Reserve and Hubei Longmen River National Forest Park will also be included in the vision planning area of the park.

The gradual expansion of Shennongjia National Park in different stages of development in The Plan is a temporary solution to the current tense relationship between people and land. In the pilot phase, the zoning of Shennongjia National Park deliberately avoided collectivized land and densely populated areas. Shennongjia Forest district has a population of nearly 80,000, and the relationship between people and land is complicated. Hongping Town and Songluo Township in the north of the park are more densely populated, with more development activities and complex land ownership, so they were partly excluded from the scope of the national park during the pilot period. Xiaguping township and the southern area of Muyu Town were designated as the national park trust area. Among them, 92.33% of the trust area is collective land, which can avoid community conflicts and financial pressure caused by land ownership. However, according to the long-term planning of Shennongjia National Park, the area of the park will be gradually expanded in the future, and the areas mentioned above with complicated land property rights will be gradually assigned to the national park, and the land ownership problem will gradually become prominent. In this context, this study not only provides a basis for making policies on the transfer or reallocation of collectively owned land in the current pilot areas but also lays a foundation for making land policies in the future expansion of national parks.

## 4. Methodology

### 4.1. Survey Questionnaire Design

On the basis of literature analysis, and in combination with the situational design questionnaire for national parks, the research contains 32 observed variables. Among them, 27 observed variables were combined into 7 latent variables (shown in Table 1). The 7 latent variables are as follows: farmers’ perception of land ecological conservation function (ECF), land environment education function (EEF), leisure and recreational function (LRF), “nest egg” function (NEF), scientific research function (SRF), land reallocation intentions (LRI), and farmers’ trust in the land management ability (TLMA) of the NPA. The other 5 observed variables covered the farmers’ preference of economic value (PEV) and the 4 characteristics of farmers: age, education, family income, and off-farm employment skills. Except for the 4 household characteristic variables, all other observed variables were measured by a 5-level Likert scale.

### 4.2. Questionnaire Distribution

Questionnaires were distributed to Xiaguping, Muyu, and Dajiuhu in Shennongjia National Park. In total, 170 questionnaires were distributed in two periods, which covered July 2019 and then July 2020. In total, 281 questionnaires on network communication were collected from December 2020 to February 2021 through the Shennongjia National Park Administration and Shennongjia poverty alleviation work QQ group. The departments of Shennongjia National Park Administration, including the Community Affairs, Policies, and Publicity and Education Division frequently communicate with community farmers in their daily work. The staff in the above-mentioned departments shared the QR code or link for the online questionnaire to the farmers when they were working in the villages, and the farmers filled in the questionnaire online and submitted it directly. In addition, as the Shennongjia National Park has a large number of farmers scattered living in the mountains more than 1000 m above sea level, it was difficult for researchers to collect questionnaires on a large scale. As a result, in our research, the staff in the Shennongjia poverty alleviation work QQ group assisted in issuing questionnaires to reduce costs and improve work efficiency. Finally, a total of 451 questionnaires were issued in this study. Among these, 121 questionnaires were collected from Xiaguping, 213 from Dajiuhu, and 117 from Muyu. In order to avoid the high redundancy in the questionnaire, the researchers only collected one questionnaire for each peasant household.

### 4.3. The Questionnaire Response

All questionnaire responses were reviewed, and invalid questionnaires were deleted. The identification of invalid questionnaires followed these criteria: first, for the network recovered questionnaires, we judged whether the questionnaires were from the same IP address according to the submission time (the questionnaires filled from the same IP address were invalided and deleted). Second, the standard deviation of all samples was tested, and each sample with a standard deviation of 0 or close to 0 was deleted. Finally, questionnaires with missing values were marked invalid, and the missing values were deleted or supplemented with the mean value, which did not exist in all questionnaires collected in this study. In accordance with the above principles, 61 invalid questionnaires were removed from the recovered questionnaires, and a total of 390 questionnaires were finally used in the research. The effective rate of the questionnaire was 86.47%.

## 5. Research Results and Analysis

### 5.1. CFA Test of Scale Reliability and Validity

#### 5.1.1. Composite Reliability and Convergence Validity

Mplus7.4 was used to perform Confirmatory Factor Analysis (CFA) for the seven latent variables in the oblique models to obtain the standard indicator loading estimate and *Squared Multiple Correlations* (*SMC*) of observe variables. Then, the Composite Reliability (*CR*) and Average of Variance Extracted (*AVE*) of latent variables were calculated. In this research, *AVE* is represented by the Convergence Validity (*CV*). Traditionally, the most common indicator of calculating the scale or testing reliability is *Cronbach’s Alpha* (*α*), where, in congeneric tests with unrelated errors, the α underestimates the reliability except for tests where τ is equivalent [34], and when the error is positively correlated, the α coefficient will overestimate the reliability. After the application of the CFA method, *CR* and *AVE* were used to calculate the internal consistency reliability [35,36]. The calculation formulas are shown in Formulas (1) and (2):

(1) $$CR=\frac{{\left(\sum \lambda \right)}^{2}}{\left[{\left(\sum \lambda \right)}^{2}+\sum \left(1-\theta \right)\right]}$$

(2) $$AVE=\;\frac{\left(\sum \lambda^{2}\right)}{\left[\left(\sum \lambda^{2}\right)+\sum \left(1-\theta \right)\right]}$$

where *λ* is the standardized factor loading estimate value, and *θ* is *SMC.* The composite reliability and convergence validity of latent variables in the scale are shown in Table 1. SPSS22.0 was used to calculate the *Cronbach’s Alpha* (*α*) of the scale.

First, the higher the *CR* value, the higher the internal consistency, where 0.7 is the acceptable threshold. Fornell and Larcker (1981) suggested a value of 0.6 or above as acceptable. Table 1 shows that the minimum *CR* value and *Cronbach’s Alpha* (α) of the latent variables in the scale are 0.853 and 0.851, respectively, which are ideal, indicating that the internal consistency of all latent variables is high. *AVE* then shows how much variation explained by potential variables is from measurement error. If *AVE* is higher, the percentage of variation explained by latent variables is higher, and the relative measurement error is smaller, which implies that the questionnaire has higher reliability and convergence validity. The ideal value should be greater than 0.5 [37], with 0.36~0.5 as the acceptable threshold. Table 1 shows that the AVE value of latent variable is at least 0.593, which is close to ideal. Finally, the standardized factor load estimation values of all observed variables in the scale were all greater than 0.7, and *SMC values* were all greater than 0.5, which was ideal.

#### 5.1.2. Discriminant Validity

There are various methods to verify Trauernichant validity, such as mean variation extraction [37], competitive model comparison [38], and the confidence interval method of correlation coefficients [39]. If the correlation between latent variables is below the absolute value of 0.7, the *AVE* method can be used for evaluation. If the correlation between latent variables is above the absolute value of 0.7, it is recommended to use the confidence interval method for estimation [40]. The correlation values of some latent variables in Table 2 were greater than 0.7, and the confidence interval method was used to test the discriminant validity of the scale by repeating the sampling 2000 times and calculating the 95% Confidence Interval (CI) of the correlation coefficient. If by calculating *Φ ± 2σ,* the bias-corrected and percentile method to calculate the correlation coefficient between the latent variables of the CI does not contain a value of 1.0, this shows good discriminant validity [31,33,39]. As shown in Table 2, the CI of the correlation coefficient calculated by the above three methods didn’t contain 1.0. Therefore, the latent variables set in this research have good discriminant validity.

### 5.2. Model Validation

#### 5.2.1. Model Fitting Degree

Mplus7.4 was used to verify the five scenario models of ECF, EEF, LRF, SRF, and NEF, and the fitting indicators are shown in Table 3. According to the judgment criteria, in five situations, except when the land has the function of environmental education (EEF), the model fitting indicator is not satisfactory, and other models all meet the fitting standard.

#### 5.2.2. Path Coefficient and Significance

Table 4 shows the non-standardized coefficient and significance of the influencing factors model of farmers’ land reallocation in different situations. Farmers’ perception of five types of land functions and the direct influence of land reallocation intention is not significant.

The above research results do not show the research conclusion that the perception of land function directly affects the land reallocation intention but indirectly affects the land reallocation intention through the mediating variable of TLMA. Hypothesis 1 and Hypothesis 2 were found to hold. Table 5 shows that the mediating effect of TLMA is obviously different in the five situations, and the situations where the mediating effect is from strong to weak are SRF (0.658), LRF (0.609), EEF (0.529), ECF (0.494), and NEF (0.437). The data show that when the land is used for scientific research, leisure, or environmental education, it is necessary for farmers to gain enough trust in the land management ability of NPA for them to leave the land. The reason is that scientific research, leisure, and environmental education in national parks are not land functions in the common sense, and farmers have limited knowledge of the land functions of the above national parks, requiring the NPA to make more efforts to gain farmers’ trust in their land management capabilities. In actuality, when the main function of land is ecological protection or living security, it is necessary to gain less trust in the land management ability of NPA, and farmers will then leave the land for it. The Chinese government initiated two nation-wide conservation policies in the late 1990s: the Natural Forest Conservation Program and the Grain-To-Green Program [41]. The Shennongjia Forest district is also involved in the two projects, and as a result, natural forest harvesting has been completely stopped. Farmers’ awareness of ecological protection has a long history, and the concept of reforestation in mountains and changing production has already taken shape. Farmers have benefited a great deal from the tourism industry, and their livelihood does not depend entirely on the consumption of natural resources. Therefore, farmers trust the government’s land management ability in terms of ecological protection and the ability to guarantee their basic livelihood.

### 5.3. Test of Moderating Effect

#### 5.3.1. Moderating Effect of PEV

The purpose of this research was to investigate whether PEV is the moderator between the perceptions of land function and land reallocation intention by using Latent Moderated Structural Equations (LMSE). LMSE model analysis results are shown in Table 5. In the scenario model of NEF, ECF, EEF, and LRF, the interaction term of PEV and land function has no significant influence on LRI, so the variable PEV has no significant moderating effect on LRI. In the scenario model of SRF, the PEV negatively affects LRI only at the significance level of 10%. The analysis results reject Hypothesis 3.

The conclusions presented by the analysis above are not consistent with the previous research [19] against the background of urbanization, which holds that the higher the PEV is, the stronger the LRI is. A study in India shows that states with more rental-market activity feature less misallocation and reallocate land more efficiently over time [42]. The Shennongjia National Park, the case of this study, is located in Shennongjia Forest district, Hubei Province. Since the implementation of the Natural Forest Conservation Program and the Grain-To-Green Program in this administrative region in the late 1990s, ecological protection policy has been put into practice for almost 20 years and has become solidified in farmers’ ideology. This land is located in a mountainous area, and the topographic and geomorphic conditions with a large slope are not suitable for large-scale urbanization development, so the land does not show economic value under the background of urbanization. It is unrealistic and difficult for farmers to obtain high economic returns through land transfer or expropriation. As a result, in the four situations where land functions are ecological conservation, “nest eggs”, leisure and recreation, and environmental education, the moderating effect of PEV on farmers’ LRI is not significant. This conclusion is in line with the conclusions of Yang et al. (2013) and Xu et al. (2014) that farmers in underdeveloped areas and distant suburbs do not have a strong perception of the economic value of land [19,29]. Under the condition that the land function is for scientific research, farmers have a low perception of the local economic value of land, which is also the actual situation in such cases, so farmers are inclined to transfer land under these circumstances. However, when the economic value of the land is high, farmers will keep the land, and the NPA must gain enough trust from farmers to improve the willingness of farmers to leave the land. This shows that when national parks realize the function of scientific research, NPA play an important role in LRI. It also implies that farmers do not quite understand and recognize the scientific research function of national parks.

#### 5.3.2. Moderating Effect of HC

Multiple group analysis is used to explore whether group variables (farmer characteristics) have the function of moderating the theoretical model. The software AMOS21.0 was used for multi-group analysis of the samples. According to age, education level, household income, and off-farm employment skills, the sample was divided into high and low groups to measure the differences in LRI between the two groups, as shown in Table 6.

The purpose of this study was to test whether the model path has unique structure invariance between different groups by conducting the test for partial invariance through AMOS21.0. According to the research literature of Wen et al. (2005), Zhao (2007), and Xu (2010), we followed the steps listed below [43,44,45].

First, the data were grouped according to the characteristics of farmers. Second, we set the two models, namely the Unconstrained Model and Structural Weights Model: the Unconstrained Model was not limited to any parameters, while the Structural Weights Model defined two groups in which the latent variable path regression coefficient was equal. The above two models form the Nested Model, and we determined the signifcance of $\Delta \chi^{2}$ in Δdf. As if $\Delta \chi^{2}$ reached a significant level (*p* < 0.01, *p* < 0.05 or *p* < 0.1), this indicated that the model path had no causal structural invariance in different groups; that is, the group variables (characteristics of farmers) had a moderating effect on the model. The model’s $\chi^{2}$/*df*, CFI, TLI, RMSEA, SRMR are basically within the ideal range, with a good fitting degree. The significance of the comparison results of the Nested Model is shown in Table 7.

By combining the path coefficient of multiple groups and data in Table 7, the following results were obtained. The unreported path coefficients were insignificant.

Age has a significant moderating effect on the scenario models of NEF, ECF, and SRF. In the NEF scenario model, the mediating effect was 0.215 in the high group and 0.656 in the low group, and the direct effect was 0.188 in the high group. The mediating effect of the ECF scenario model was 0.224 in the high group and 0.521 in the low group, and the direct effect was 0.159 in the low group. The mediating effect of the SRF scenario model was 0.372 in the high group and 0.649 in the low group. It can be seen that the LRI of the low group is stronger. Against the background of China’s current urbanization, the rural hollowing out phenomenon is becoming increasingly serious—the elderly and children were left behind on the land, and the young migrant workers were more likely to give up land—especially when the land functions only for nest eggs, ecological conservation, and scientific research, land does not directly bring economic benefits. The low age group of farmers were not attracted by land, and they were more likely to give up land. On the other hand, due to the lack of off-farm skill learning ability, high group farmers were more inclined to stay on the land to obtain basic security. When the land function is leisure and recreation and environmental education, age does not have a significant moderating effect on the model. In the LRF scenario model, the mediator effect of the low group was 0.791, the direct effect was 0.298, and the mediating effect of the high group was 0.459. In the EEF scenario model, the mediating effect of the low group was 0.571, the mediating effect of the high group was 0.324, and the direct effect was 0.422. As recreation and ecological education can bring direct economic benefits to local communities and promote community development, communities have a higher degree of support for the construction of national parks, which is reflected in the willingness of farmers to hand over their land to the NPA regardless of their age level.

Education shows a very significant moderating effect on the scenario models of NEF, LRF, and SRF. Forest persistence was positively affected by increases of basic education percentage [46]. In the NEF scenario model, the direct and mediating effects of the low group are significant, the sum of which is 0.410, while the mediating effects of the high group are only slightly significant, at 0.644. In the LRF scenario model, the LRI of the low group is only affected by the mediation path, with a mediation effect of 0.436. The direct and mediating effects of high group farmers on LRI were 0.335 and 0.683, respectively, and the sum of the effects was 1.018. In the SRF scenario model, the LRIs of farmers in the low group and the high group were only affected by the mediation path, and the mediating effect was 0.319 and 0.703, respectively. Thus, it can be seen that farmers with a higher education level have a better understanding of the functions of land for nest eggs, leisure and recreation, and scientific research in national parks and are more willing to leave the land in order to realize these functions. In addition, they will consider whether the NPA has enough ability to manage these lands well when they leave the land.

Family income has a significant moderating effect on the scenario models of NEF, ECF, and SRF. In the NEF scenario model, the direct effect of the low group was 0.230, the mediating effect was 0.545, and the sum of the effects was 0.775. In the high group, only the mediating effect was significant, at 0.315. In the ECF scenario model, farmers’ LRIs were only affected by the mediation path, and the mediating effects of the low group and the high group were 0.565 and 0.223, respectively. Thus, when the land function is for nest eggs and ecological conservation, the added value of land cannot be reflected, and the direct benefits brought by it are low. Farmers in the low-income family group are more inclined to give up the land and seek for a more sustainable kind of livelihood. In the SRF scenario model, farmers’ cognition of the land’s scientific research function can only affect the LRI through the mediating variable, and the mediating effects of the low group and the high group are 0.586 and 0.662, respectively. This reveals that farmers will give up their land only if they have enough trust in the NPA, and farmers in high income families are more willing to give up land. This also shows that farmers do not quite understand the scientific research function of national parks, and the NPA needs to strengthen the publicity of the scientific research function of national parks.

Off-farm employment skills have a significant moderating effect on the scenario models of NEF, LRF, and EEF. The mediating effect and direct effect of the EEF scenario model were both present. The mediating effect of the low group was 0.411, the direct effect was 0.342 and the sum was 0.753. In the high group, the mediating effect was 0.523, the direct effect was 0.177, and the sum was 0.700. The sum of the two groups of effects was basically the same. When the land function was environmental education, there was no significant difference in LRI, regardless of off-farm employment skills. However, in the NEF scenario model, farmers only generated land reallocation intentions through the mediation path, with the mediating effect of 0.550 in the low group and 0.391 in the high group. In the LRF scenario model, the LRI of the low group was not significant, while the LRI was generated by the high group only through the mediating path, with a mediating effect of 0.583. This indicates that when the land function is for leisure and recreation and life security, farmers will only give up the land if they have enough trust in the NPA. At the same time, when the land function is leisure and recreation, farmers with off-farm employment skills are more likely to give up the land. These farmers will make use of their off-farm employment skills to benefit by participating in leisure and recreation, such as catering, accommodation, and other reception businesses or providing guide services. When the land function is life security, farmers without off-farm employment skills are more likely to give up their land. This may not be consistent with common sense, but it is common practice in China. The natural resources of the protected land are strictly protected, and the function of farmers to ensure a minimum standard of living through farming activities on the land cannot be guaranteed in some core protected areas. As a result, a lack of off-farm employment skills means that farmers cannot get income from the land, which will only aggravate their poverty level. So, farmers without off-farm employment skills are more likely to give up their land. The Chinese government is addressing the above problem through the relocation of poverty alleviation, ecological migration, and other measures. There is a robust negative effect of land reallocation on the amount of time that villagers devote to off-farm work [47].

### 5.4. Characteristics of Farmers and Compensation Form of Land Reallocation

In this study, three observed variables (A30, A31, and A32) were used to measure the latent variable LRI. A32 is a five-level quantification of the degree to which “I prefer livelihood security to monetary compensation in terms of land reallocation”. The results of the comparative mean analysis and the ANOVA test are shown in Table 8.

The data showed that the quantitative score of observation variable A32 was 4.09, and farmers were more willing to get livelihood compensation. At the same time, off-farm employment skills and household income do not have a significant impact on the willingness to take livelihood compensation; age and education level had a significant influence on the willingness to take livelihood compensation among the groups, and farmers with an advanced age and lower education level were more likely to be eligible for livelihood compensation.

## 6. Conclusions

First, farmers’ cognition of the land functions in national parks affects their land reallocation intention through mediation variables, and farmers’ trust in the land management ability of NPA is a complete mediator between farmers’ land function cognition and their willingness to leave the land. In the five land function scenario models of scientific research, leisure and recreation, environmental education, ecological protection, and livelihood security, the mediating effect value of the variable of farmers’ trust in NPA’s land management ability decreased gradually. The results showed that rural households did not understand the non-conventional functions of national parks, such as scientific research, recreation, and environmental education. Therefore, when land is used for scientific research, recreation, and environmental education, the NPA needs to gain sufficient trust from farmers in order to improve farmers’ willingness to leave land.

Second, PEV has no significant positive moderating effect on the relationship between land functions (ECF, EEF, NEF, LRF, SRF) and land reallocation intention (LRI). According to the actual situation in the case study, if farmers perceive that the economic value of the land is low in the scenario of SRF, they are inclined to transfer the land. However, when the economic value of the land is higher, the farmers tend to reserve the land. At this time, the NPA must gain high trust from farmers to promote the improvement of farmers’ willingness to leave the land. PEV has a negative moderating effect on the relationship between the land function of scientific research and land reallocation intention. This also reveals that farmers’ cognition of the scientific research function of national parks is insufficient.

Third, the moderating effects of peasant household characteristics on different situation models are not the same. ① Age has a significant moderating effect on the scenario models of NEF, ECF, and SRF. The land reallocation intention was stronger among the farmers of the younger age group. This is supported by the research of Hu et al. (2018) [48] and Tang et al. (2014) [49]. ② Education shows a very significant moderating effect on the scenario models of NEF, LRF, and SRF. The willingness of highly educated farmers to leave their land is stronger, and with the improvement of education level, the willingness of highly educated farmers to leave their land will increase with the degree of trust in the land management ability of NPA. This conclusion was confirmed in the study of Tang et al. (2014) [49]. ③ Family income has a significant moderating effect on the scenario models of NEF, ECF, and SRF. Due to the strict ecological protection restrictions, the livelihood of farmers in the low-income family group is not sustainable, and they are more inclined to give up their land. This is consistent with the research conclusion of Hu et al. (2018) [48]. ④ Off-farm employment skills have a significant moderating effect on the scenario models of NEF, LRF, and EEF. When the land function is leisure and recreation, farmers with off-farm employment skills are more inclined to give up their land. When the land function is life security, farmers without off-farm employment skills are more likely to give up their land.

Fourth, compared with material or monetary compensation, land-losing farmers are more willing to receive livelihood compensation, and the less educated and older farmers are more willing to receive livelihood compensation after land reallocation.

Finally, in the context of nature conservation and urbanization in China, there are differences in farmers’ willingness to leave the land. ① Against the background of urbanization, when the economic value of land is high, farmers are willing to leave the land to obtain compensation [16], but against the background of nature protection, the economic value of land has no significant moderating effect on the willingness of farmers to leave the land. ② The management ability of NPA is a completely mediating factor for the peasants’ land reallocation intention, but the government’s land management ability is rarely mentioned in the study of land reallocation intention against the background of urbanization. ③ Farmers without off-farm employment skills in nature reserve communities were more likely to give up their land, while farmers without off-farm employment skills were not found to be likely to do so in the context of urbanization [14,50]. The reason is that, against the background of nature protection, the land use mode and intensity are strictly restricted, and the minimum subsistence security function of the land cannot be ensured, so the farmers have to give up the land to find another livelihood. Farmers with off-farm employment skills can benefit from participation in recreational and ecotourism operations, so they tend to stay on their land.

## 7. Applications

According to the Guidelines, national parks are divided into two functional zones: the Strictly Protected Zone (SPZ) and Generally Controlled Zone (GCZ). Of these, the SPZ is devoted to carrying out ecological protection and the scientific research function of the land, while the GCZ can be further refined to consider land functions such as leisure and recreation, environmental education, and living guarantees to promote community development. The land function situations in this study can be combined with the functional zoning of national parks in the Guidelines. In order to adhere to the principle of ecological protection first, the collective land in the Strict Protection Zones needs to be nationalized. In order to protect the rights and interests of community development, collective land in zjr GCZ need not be fully expropriated, but the mode and intensity of land use need to be limited and can be transferred to the park management agency or a third party when necessary. In any type of land function scenario, the NPA needs to deal with Collective Land Ownership. To prevent community conflicts, this study proposes the following collective land management recommendations.

The capacity for the building of national parks to manage natural resources such as land needs to be strengthened. The construction of park natural resource management capacity is the key to realize the strict collection of collective land in protected areas and the transfer of collective land in general controlled areas.

A national park community communications and support department should be established within the NPA with the purpose of strengthening the communication between the national park and the community and popularizing the background and significance of the park construction in the community to improve the cognition of the community farmers of the basic functions of the national park. The key points of the work are to strengthen farmers’ cognition of the scientific research function of national parks, to increase communication with young and highly educated people with off-farm employment skills and a low living guarantee whose land is located in the general control zone in order to gain their trust in the NPA, to help community farmers to improve their off-farm employment skills and raise their household income, and finally to increase investment in basic community education needs. The NPA should work with schools in the compulsory education stage in the park community to carry out national park education and improve young people’s awareness of the functions of national parks to foster a positive emotional connection with national parks.

Attention should be paid to the compensation method of community farmers’ land reallocation. In the process of acquiring land control rights, the NPA should give preference to livelihood compensation for farmers who are older or less educated, so that farmers can acquire lasting “nest eggs”. In the process of the ecological migration of the original Dajiuhu Village in Shennongjia National Park, the NPA leased the collective land located in SPZ (Dajiuhu Wetland) for 30 years. For the Dajiuhu immigrants who have moved to Pingqian Town, the NPA has guided the immigrants to engage in accomodation, catering, and other service industries in Pingqian Town through systematic tourism training. The NPA has also given preferential interest rates for loans to the accomodation operators of ecological migrants. At present, Pingqian Town is another important tourist node in Shennongjia National Park besides Muyu Town. It has been proved that Shennongjia National Park pays attention to farmers’ demands for livelihood compensation in the disposal of the Collective Land Ownership of protected land and obtains community support through diversified land compensation methods, realizing a win–win situation of ecological protection and community development.

## 8. Limitation and Prospect

The statistical data of the samples in this research show that farmers with a college education or a graduate degree and young farmers account for a large proportion of the samples, which may be due to the large proportion of questionnaires collected through the Internet. The penetration rates of smart phones and the Internet are higher among farmers who are young or have a high education. Limited by objective factors, the sample data collection rate from the field household survey was not high, which is the limitation of this research.

At present, among the 10 national parks in China, the collective land of Sanjiangyuan, Qilian Mountain, Northeast Hubao, Puda Cuo, and Hainan tropical rain forest accounts for less than 20%, and the largest proportion of the collective land area is 80.73% in Qianjiangyuan, followed by 74.74% in Wuyi Mountain, 64.42% in Nanshan Mountain, and 28.59% in Giant Panda. In this case, the collective land of Shennongjia National Park (including the area of the trust area) accounts for 23.2%, which is at a medium level. The research on the difference of the reallocation intention of collective land in different types and regions of national parks can be taken as a future research direction. Especially after the end of the national park system pilot project, China’s national parks will continue to expand. By then, the comparative study on the land reallocation intentions of community farmers of collective land in national parks in southern, northern, and central China will provide a scientific basis for differentiated land ownership policies in protected areas.

## Figures and Tables

**Table 1:** The *CR* and *AVE* of the scale based on CFA.

Latent Variable	Items		*Std* (*λ*)	*SMC* (*θ*)	*Cronbach’ Alpha* (*α*)	*CR*	*AVE*
Ecosystem Conservation Function (ECF)	A6	Forest, grassland, and other land ecosystems are the main ecosystems on the earth.	0.714	0.509	0.930	0.930	0.655
	A7	Humans are not the only owners of the land. The land is also home to plants and animals.	0.794	0.631			
	A8	Land is the foundation of the growth of all living things and the space carrier of natural ecosystem.	0.870	0.756			
	A9	Land is the carrier of traditional culture, and the destruction of land ecology will affect the inheritance of traditional culture.	0.753	0.567			
	A10	National parks are nature protected areas, and their land use should be based on ecological protection.	0.859	0.738			
	A11	The land can be used for vegetation growth to regulate climate, purify the environment, and reduce noise pollution.	0.832	0.692			
	A12	The conservation of the land ecology in national parks preserves development opportunities for future generations.	0.832	0.691			
Nest Eggs Function (NEF)	A13	Land can provide a minimum livelihood for family members.	0.723	0.523	0.857	0.862	0.678
	A14	Land gives family members pension security.	0.874	0.764			
	A15	Land can provide unemployment insurance for family members.	0.864	0.746			
Leisure & Recreation Function (LRF)	A16	National park land is the carrier of natural and cultural tourism resources.	0.786	0.617	0.884	0.880	0.647
	A17	National park land provides space for human recreation and leisure activities.	0.752	0.565			
	A18	The recreation and leisure industry of a national park can provide employment chances for the local community and promote incomes of local families.	0.828	0.685			
	A19	The development of national park tourism industry can activate tradition culture, and the tradition culture can be inherited.	0.848	0.719			
Scientific Research Function (SRF)	A20	The land ecosystem is the vital research subject in the science area.	0.784	0.615	0.881	0.876	0.703
	A21	The land science research works try to balance the relationship between development and conservation and provide the basis for wise land use.	0.821	0.674			
	A22	Land science knowledge is the significant content of environment education.	0.905	0.819			
Environmental Education Function (EEF)	A23	Environment education in a national park can enable people to understand the land ecosystem and increase environment protect knowledge.	0.900	0.810	0.917	0.917	0.786
	A24	Environment education in a national park can promote people’s awareness of environment protection.	0.875	0.766			
	A25	Environment education in a national park can cause people to engage in environment protection behavior.	0.884	0.782			
Trust in Land Management Ability (TLMA)	A26	The national park service knows better how to preserve the land ecological environment.	0.797	0.634	0.851	0.853	0.593
	A27	The national park service knows better how to wisely explore and use land.	0.812	0.659			
	A28	The national park service can obtain land ownership with important ecological functions.	0.752	0.565			
	A29	The national park service has the power to regulate the use of all land within the park.	0.716	0.512			
Land Reallocation Intention (LRI)	A30	If monetary compensation is reasonable, I am willing to transfer land property to the national park.	0.799	0.638	0.858	0.858	0.669
	A31	If national parks provide alternative livelihoods, I am willing to transfer land property to the national park.	0.875	0.765			
	A32	I prefer livelihood security to monetary compensation in terms of land reallocation.	0.777	0.604			

**Table 2:** Discriminate validity results.

Pairs of Correlation			Estimate	S.E.	*Φ* ± *2σ*		95% CI					
							Bias-Correct			Percentile		
					Lower	Upper	Lower	Upper	*p*	Lower	Upper	*p*
NEF	↔	LRF	0.513	0.052	0.409	0.617	0.362	0.654	0.001	0.351	0.649	0.001
NEF	↔	SRF	0.448	0.055	0.338	0.558	0.295	0.597	0.001	0.286	0.587	0.001
NEF	↔	EEF	0.485	0.052	0.381	0.589	0.341	0.628	0.001	0.327	0.621	0.001
NEF	↔	TLMA	0.495	0.054	0.387	0.603	0.342	0.626	0.001	0.340	0.625	0.001
NEF	↔	LRI	0.502	0.053	0.396	0.608	0.357	0.644	0.001	0.357	0.644	0.001
NEF	↔	ECF	0.348	0.058	0.232	0.464	0.158	0.521	0.001	0.158	0.521	0.001
LRF	↔	SRF	0.866	0.024	0.818	0.914	0.788	0.930	0.001	0.779	0.924	0.001
LRF	↔	EEF	0.887	0.020	0.847	0.927	0.817	0.940	0.001	0.813	0.938	0.001
LRF	↔	TLMA	0.766	0.034	0.698	0.834	0.671	0.850	0.001	0.660	0.845	0.001
LRF	↔	LRI	0.710	0.039	0.632	0.788	0.587	0.812	0.001	0.586	0.811	0.001
LRF	↔	ECF	0.765	0.031	0.703	0.827	0.605	0.877	0.001	0.607	0.878	0.001
SRF	↔	EEF	0.850	0.024	0.802	0.898	0.769	0.921	0.001	0.762	0.915	0.001
SRF	↔	TLMA	0.730	0.037	0.656	0.804	0.603	0.845	0.000	0.584	0.834	0.000
SRF	↔	LRI	0.592	0.047	0.498	0.686	0.437	0.718	0.001	0.437	0.716	0.001
SRF	↔	ECF	0.734	0.033	0.668	0.800	0.580	0.830	0.002	0.597	0.839	0.001
EEF	↔	TLMA	0.782	0.031	0.720	0.844	0.670	0.868	0.001	0.660	0.859	0.001
EEF	↔	LRI	0.709	0.038	0.633	0.785	0.584	0.818	0.001	0.579	0.815	0.001
EEF	↔	ECF	0.688	0.036	0.616	0.760	0.532	0.803	0.001	0.534	0.804	0.001
TLMA	↔	LRI	0.819	0.032	0.755	0.883	0.713	0.898	0.001	0.701	0.896	0.001
TLMA	↔	ECF	0.603	0.045	0.513	0.693	0.438	0.741	0.001	0.437	0.740	0.001
LRI	↔	ECF	0.547	0.048	0.451	0.643	0.367	0.679	0.001	0.376	0.685	0.001

**Table 3:** Test of fitting degree of SEM.

Fit Indicator	Criteria	Scenario Model				
		ECF	NEF	LRF	SRF	EEF
X^2^	The smaller, the better	184.999	70.389	116.632	85.064	156.971
$\frac{X^{2}}{df}$	<3	2.569	2.271	2.926	2.744	5.064
CFI	≥0.9	0.960	0.977	0.962	0.970	0.940
TLI	≥0.9	0.950	0.966	0.948	0.957	0.913
RMSEA	≤0.08	0.074	0.066	0.081	0.078	0.118
SRMR	≤0.08	0.050	0.047	0.044	0.041	0.054

**Table 4:** Unstandardized path coefficients and significance of the model.

Scenario	Path			Estimate (Regression Weight)	S.E.	Est./S.E.	Two-Tailed *p*-Value
ECF	TLMA	←	ECF	0.495	0.109	4.451	***
	LRI	←	TLMA	0.998	0.156	6.415	***
	LRI	←	ECF	0.134	0.096	1.399	0.162
NEF	TLMA	←	NEF	0.429	0.093	4.635	***
	LRI	←	TLMA	1.018	0.158	6.426	***
	LRI	←	NEF	0.138	0.095	1.455	0.146
LRF	TLMA	←	LRF	0.690	0.109	6.312	***
	LRI	←	TLMA	0.882	0.187	4.718	***
	LRI	←	LRF	0.240	0.172	1.397	0.162
SRF	TLMA	←	SRF	0.616	0.095	6.460	***
	LRI	←	TLMA	1.068	0.214	5.003	***
	LRI	←	SRF	−0.015	0.175	−0.086	0.932
EEF	TLMA	←	EEF	0.603	0.107	5.637	***
	LRI	←	TLMA	0.877	0.195	4.502	***
	LRI	←	EEF	0.206	0.160	1.292	0.196

* *p* < 0.1, ** *p* < 0.05, *** *p* < 0.01.

**Table 5:** Path coefficient and significance of PEV moderating effect model.

Scenario model	Path			Estimate (Regression Weight)	S.E.	Est./S.E.	Two-Tailed *p*-Value
ECF	LRI	←	TLMA	0.977	0.147	6.628	***
	LRI	←	ECF	−0.084	0.262	−0.322	0.747
	**LRI**	←	ECF * PEV	−0.034	0.036	−0.929	0.353
	LRI	←	PEV	0.187	0.280	0.669	0.504
	TLMA	←	ECF	0.519	0.111	4.691	***
NEF	LRI	←	TLMA	0.940	0.160	5.883	***
	LRI	←	NEF	0.023	0.117	0.199	0.843
	**LRI**	←	NEF * PEV	0.020	0.083	0.237	0.813
	LRI	←	PEV	0.323	0.187	1.731	0.083 (*)
	TLMA	←	NEF	0.449	0.094	4.783	***
LRF	LRI	←	TLMA	0.873	0.182	4.796	***
	LRI	←	LRF	0.049	0.359	0.138	0.891
	**LRI**	←	LRF * PEV	−0.047	0.042	−1.119	0.263
	LRI	←	PEV	0.183	0.368	0.498	0.618
	TLMA	←	LRF	0.711	0.108	6.567	***
SRF	LRI	←	TLMA	1.032	0.205	5.042	***
	LRI	←	SRF	−0.380	0.312	−1.217	0.224
	**LRI**	←	SRF * PEV	−0.068	0.039	−1.747	0.081 (*)
	LRI	←	PEV	0.457	0.355	1.287	0.198
	TLMA	←	SRF	0.636	0.096	6.651	***
EEF	LRI	←	TLMA	0.846	0.185	4.585	***
	LRI	←	EEF	0.070	0.217	0.321	0.748
	**LRI**	←	EEF * PEV	−0.030	0.035	−0.868	0.385
	LRI	←	PEV	0.190	0.226	0.839	0.401
	TLMA	←	EEF	0.627	0.111	5.647	***

* *p* < 0.1, ** *p* < 0.05, *** *p* < 0.01.

**Table 6:** Grouping according to sample characteristics.

Characteristics		Grouping Criterion	Low Group	High Group
Personal characteristics	Age	The low group is under 25 years of age; age 25 and above is the high group.	247	143
	Education	Tertiary education and above are in the high group; below college education level is the low group.	136	254
Household	Household income	Ministry of Agriculture: In 2017, the per capita disposable income of rural residents is about 13,000 yuan. Based on the three members of a nuclear family, incomes of 40,000 yuan and above are classified as the high group. The low group earns 40,000 yuan or less.	202	188
	Off-farm employment skills	Non-agricultural employment skills were sorted into the high group; skills without off-farm employment were sorted into the low group.	121	269

**Table 7:** The significant of Nested Model comparisons (*p*-value).

	Characteristics	Age	Education	Income	Off-Farm Skill
Scenario					
NEF		0.009 (***)	0.003 (***)	0.006 (***)	0.009 (***)
ECF		0.013 (**)	0.132	0.000 (***)	0.326
LRF		0.213	0.002 (***)	0.256	0.024 (**)
SRF		0.078 (*)	0.001 (***)	0.042 (**)	0.230
EEF		0.408	0.406	0.165	0.094 (*)

* *p* < 0.1, ** *p* < 0.05, *** *p* < 0.01.

**Table 8:** The compensation for land reallocation according to the characteristics of farmers.

Characteristics		Mean	N	Ratio (%)	Standard Deviation	ANOVA Intergroup Significance
Age	18–25	3.95	247	47.18	1.023	0.024 **
	26–35	4.24	51	9.74	1.051	
	36–45	4.41	62	11.79	1.024	
	46–55	4.37	22	4.10	0.806	
	>56	4.5	8	1.54	0.837	
Education	Without education	4	4	0.77	1	0.019 **
	Primary school	4.57	9	1.79	0.787	
	Junior high school	4.36	48	9.23	0.99	
	High school	4.36	74	14.10	0.93	
	Junior college and above	3.95	254	48.46	1.045	
Off-farm employment skills	No	4.13	121	23.08	1.019	0.903
	Yes	4.08	269	51.28	1.034	
Income (yuan per year)	3000–5000	4.06	48	9.23	1.068	0.559
	5000–10,000	4.1	55	10.51	1.114	
	10,000–20,000	3.88	66	12.56	1.033	
	20,000–30,000	4.17	32	6.15	1.007	
	>30,000	4.16	188	35.90	0.994	
total		4.09	390	100	1.026	--

* *p* < 0.1, ** *p* < 0.05, *** *p* < 0.01.

## Data Availability

Not applicable.
